# Mitochondrial Abundant Heat Soluble (MAHS) Protein Expression Modulates Metabolic Dynamics in Human Adipose-Derived Stem Cells

**DOI:** 10.3390/ijms27146289

**Published:** 2026-07-15

**Authors:** Jordan L. Rolsma, Julieanna Li, Ruthia Soh, Connor Nieh, Bryce Yeh, William Darch, Nicole zur Nieden, Joshua T. Morgan

**Affiliations:** 1 Department of Bioengineering, University of California, Riverside, CA 92521, USA; 2 Department of Molecular, Cell & Systems Biology, University of California, Riverside, CA 92521, USA; 3 Stem Cell Center, College of Natural and Agricultural Sciences, University of California, Riverside, CA 92521, USA

**Keywords:** mitochondria, tardigrade, mitochondrial heat-soluble

## Abstract

Adipose-derived stem cells (ASCs) possess substantial regenerative potential, with lineage fate tightly coupled to metabolic dynamics. In prior work, we found that expression of the tardigrade-derived protein Mitochondrial Abundant Heat Soluble (MAHS) increased stress tolerance and promoted ASC osteogenic differentiation over adipogenic differentiation, although the mechanism remained unclear. Here, we leverage transcriptomic and functional assays to investigate how MAHS expression influences ASC metabolism. Transcriptomic profiling revealed incomplete adipogenic activation alongside upregulation of ossification-associated genes (e.g., *SERPINF1*), as well as broader changes in metabolic, Wnt, and YAP signaling pathways consistent with a shift toward osteogenic programming. Functionally, MAHS-expressing ASCs exhibited increased matrix calcium deposition in osteogenic culture, as well as decreased ATP and cAMP levels during basal culture. Further, treatment with forskolin increased cAMP levels, while forskolin or Compound C increased lipid accumulation in MAHS-expressing ASCs. Together, these findings suggest that MAHS expression perturbs metabolic dynamics and signaling networks in ASCs and may contribute to impaired adipogenesis and reinforcement of osteogenic bias. More broadly, this work highlights potential regulatory points that could be targeted to restore lineage balance in metabolically disrupted ASCs.

## 1. Introduction

Human adipose-derived stem cells (ASCs) are increasingly used in regenerative tissue repair, recognized for their multilineage differentiation capabilities, promotion of tissue repair, and beneficial immunomodulatory effects [1]. However, ASC therapeutic efficacy is often limited by various environmental and internal stressors, including cryopreservation stress, injection shear forces, and nutrient deprivation within damaged tissue microenvironments [2,3,4,5,6]. These stressors can reduce ASC viability, necessitating higher cell therapeutic doses or repeated treatment administrations that pose clinical application challenges.

One useful approach to enhancing ASC viability is transgenic expression of cytoprotective proteins. Mitochondrial Abundant Heat Soluble (MAHS), a protein derived from the extremophilic animal species *Ramazzottius varieornatus*, has been shown to confer enhanced survival characteristics to HEp-2, endothelial, and ASC lines under various stress conditions [7,8,9]. In prior work, we demonstrated that MAHS-expressing ASCs experienced increased viability during chronic dimethyl sulfoxide (DMSO) exposure, shear stress during injection simulations, and metabolic stress during nutrient depletion [8]. However, we also observed altered differentiation behavior. Specifically, MAHS-expressing ASCs cultured in osteogenic media showed increased alkaline phosphatase activity, and cells cultured in adipogenic media reduced BODIPY-positive lipid accumulation, indicative of enhanced early osteogenesis and impaired adipogenesis, respectively [8]. Importantly, further studies demonstrated that robust lipid droplet formation could be induced with increased glucose supplementation, suggesting that adipogenic differentiation is altered, but not abolished, by MAHS expression.

Because MAHS is an exogenous protein with no known human homolog, its relevance lies not in modeling endogenous human physiology, but in its potential use as a cell engineering tool to improve the stress tolerance of therapeutic cells. As the differentiation changes described above will influence potential therapeutic applications, understanding how MAHS influences ASC differentiation is critical for evaluating its broader implications in regenerative medicine as a cell engineering strategy. In this study, we aim to elucidate the molecular and cellular pathways influenced by exogenous MAHS expression in ASCs, with the goal of identifying the mechanisms responsible for the observed differentiation phenotype to aid in the rescue of adipogenic capacity. As adipogenic and osteogenic differentiation are governed by highly interconnected signaling networks, interested readers are directed to detailed schematics and mechanistic overviews of these processes available in comprehensive reviews [10,11,12], with key aspects highlighted here.

Metabolism is a critical component of differentiation and maintenance of stemness in ASCs [13]. One key metabolic regulator is AMP-activated protein kinase (AMPK), which influences ASC fate by promoting and inhibiting catabolic and anabolic pathways, respectively [14]. AMPK activation can support osteogenesis while simultaneously suppressing adipogenesis through modulation of mTOR and other downstream lineage commitment markers [15]. Another key metabolic sensor, cyclic adenosine monophosphate (cAMP), has important implications in early and late stages of adipogenic and osteogenic differentiation. Notably, low initial cAMP levels are conducive to osteogenesis, as its upregulation during the early stages of osteogenic differentiation has been shown to downregulate osteogenic markers such as *RUNX2*, *SP7*, and *IBSP* [16]. However, late-stage cAMP upregulation promotes osteocalcin production and mineralization, suggesting that temporal cAMP regulation is necessary for optimal osteogenic differentiation [16]. Conversely, elevated cAMP can enhance adipogenesis and adipocyte maturation through activation of exchange protein directly activated by cAMP (Epac) and cAMP-dependent protein kinase (PKA) [17], as well as through cyclin E-mediated sensitization of growth-arrested ASCs to apoptosis induced by DNA damage [18]. These observations highlight the dual and stage-dependent effects of cAMP in stem cell fate decisions, with AMPK activity acting in opposition to cAMP signaling in the context of energetic stress. This emphasizes the necessity of tightly controlled cAMP regulation to ensure proper differentiation and maturation processes. cAMP levels can be experimentally modulated in ASCs using well-known stimulators such as the adenylyl cyclase activator forskolin (FSK) [16].

Exogenous expression of MAHS may have unspecified effects in ASCs. Prior work has hypothesized that MAHS may embed within the mitochondrial membrane of other human cell types [7,19]. This may disrupt cellular mitochondrial membrane potential and have further effects on downstream metabolic pathways pertaining to the production of ATP and cAMP [20,21]. If present, these metabolic changes may be involved in the altered differentiation potential of ASCs, as metabolism has been shown to play a critical role in expression of genes guiding osteogenic and adipogenic differentiation (e.g., *ALPL* and *CEBPA*) [22,23,24,25,26].

Herein, we investigate the impact of MAHS expression on metabolic dynamics in ASCs, with particular focus on its role in modulating pathways involved in energy regulation and lineage commitment. Specifically, we perform transcriptomic analysis via bulk RNA sequencing and a series of targeted functional assays assessing mitochondrial membrane potential, intracellular ATP levels, and cAMP signaling. We also examine the potential for rescue of the impaired adipogenic phenotype in MAHS-expressing ASCs using FSK and Compound C.

## 2. Results

### 2.1. MAHS Expression Broadly Alters ASC Transcriptomics

**Figure 1:**
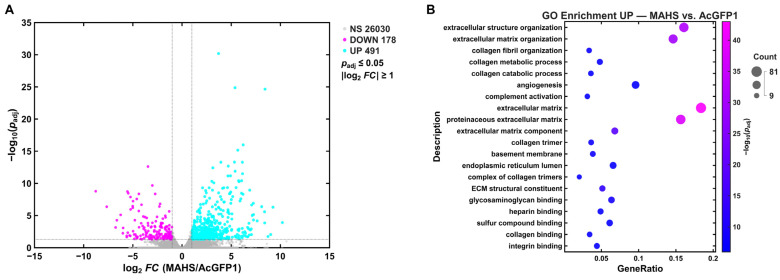
Differentially expressed gene (DEG) and Gene Ontology (GO) analysis. (**A**) Volcano plot of DEGs (cyan = significantly upregulated, magenta = significantly downregulated, gray = not significant (NS)). Significance thresholds were established at adjusted *p*-value (*p*_adj_) < 0.05 and |log2fold change (FC)| > 1, indicated by dashed lines. Display is truncated at −15 and 15 log_2_FC for clarity. (**B**) GO-BP enrichment of upregulated gene set, top 20 GO-BP terms shown. Dot size and color indicate number of genes in each category and *p*_adj_, respectively. GeneRatio indicates the fraction of genes in the category relative to total genes. “Extracellular Matrix” was abbreviated to ECM in one instance to prevent label truncation.

To address the broad question of whether MAHS functions as a link between metabolic regulation and lineage commitment, we began with a discovery-based approach. Using ASCs previously validated for MAHS expression [8], we performed RNA sequencing to identify differentially expressed genes (DEGs) relative to AcGFP1-transgenic controls. DEG analysis identified 491 significantly upregulated genes and 178 significantly downregulated genes in MAHS-expressing ASCs relative to AcGFP1-transgenic ASCs under routine culture conditions (Figure 1A). Meanwhile, Gene Ontology Biological Process (GO-BP) enrichment analysis of the upregulated gene set identified 313 significantly enriched biological processes encompassing 312 unique genes (*p*_adj_ < 0.05) (Figure 1B); in contrast, no significantly enriched BP terms were identified with the downregulated gene set (*p*_adj_ < 0.05). These findings demonstrate that MAHS expression broadly impacts cell phenotype, with upregulation in extracellular matrix and collagen construction, and organization and downregulation in anion transport, cation homeostasis, and membrane permeability.

**Figure 2:**
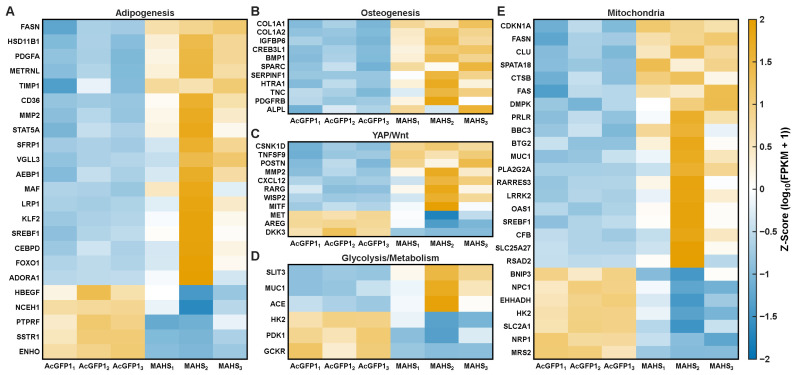
Heatmap of differentially expressed genes identified by RNA sequencing between AcGFP1- and MAHS-expressing ASC samples for gene sets (**A**) Adipogenesis (**B**) Osteogenesis (**C**) YAP/Wnt (**D**) Glycolysis/Metabolism (**E**) Mitochondria. Gene set sources are defined in the main text. Gene expression values were log_10_(FPKM + 1)-derived and z-score-normalized. Each column represents a biological replicate, and each row represents a gene that was significantly differentially expressed (*p*_adj_ < 0.05) and is associated with adipogenic or osteogenic differentiation, YAP/Wnt signaling, or metabolic or mitochondrial regulation. Significantly regulated genes that appeared in multiple gene sets are shown in each set.

We further wished to assess gene expression changes relevant to the observed phenotypes. To explore the transcriptional mechanisms underlying the reduced adipogenic potential observed in MAHS-expressing ASCs, we compared our DEG list against a curated set of adipogenesis-associated targets from the Adipose Tissue Development list in the Harmonizome GeneRIF Biological Term Annotations dataset [27,28]. This comparison revealed 16 significantly upregulated genes out of 159, suggesting that many aspects of adipogenic programming remain transcriptionally active (Figure 2). For instance, *HSD11B1* was strongly induced (log_2_FC = 4.1), consistent with enhanced local glucocorticoid synthesis, a key driver of adipogenesis [29]. *ADORA1* (log_2_FC = 3.4), a promoter of adipogenic signaling via cAMP, and *SREBF1* (log_2_FC = 1.7), a transcriptional factor associated with lipogenesis, were also elevated [30,31]. Notably, *FOXO1* (log_2_FC = 5.5) and *SFRP1* (log_2_FC = 7.3) were among the most strongly upregulated genes. *FOXO1* can increase insulin sensitivity and transcriptionally repress other adipogenic genes such as *PPARG* in an unphosphorylated state [32,33], while supporting osteogenesis [34]. The strong induction of *SFRP1*, a Wnt signaling inhibitor and context-dependent promoter of adipogenesis, may indicate a compensatory mechanism to anti-adipogenic signaling [35]. In contrast, only four adipogenesis-associated genes were downregulated. *SSTR1* (log_2_FC = −4.1) modulates insulin-dependent endocrine signaling and lipid metabolism, and reduced expression may suppress hormonal adipogenic signaling cues [36]. *ENHO* (log_2_FC = −5.2), meanwhile, influences energetic and mitochondrial homeostasis and adipogenic differentiation, and its strong suppression may indicate altered lipid metabolism [37]. *PTPRF* (log_2_FC = −1.2) and *HBEGF* (log_2_FC = −1.8), implicated in the regulation of extracellular insulin and paracrine signaling, respectively, were also reduced [38,39].

**Figure 3:**
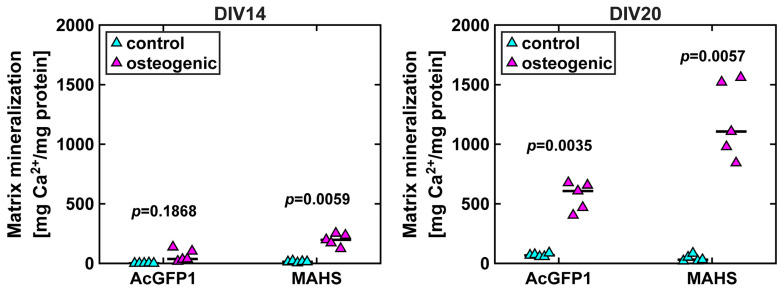
Determination of matrix calcium content. Quantification of calcium content in AcGFP1- and MAHS-expressing osteogenically differentiated ASCs (Brown-Forsythe and Welch ANOVA tests with Dunnett’s post hoc test, *n* = 5) on differentiation day (DIV) 14 and 20. *p* = 0.02, AcGFP1 osteogenic vs. MAHS osteogenic DIV14; *p* = 0.0405, AcGFP1 osteogenic vs. MAHS osteogenic DIV20. Black lines represent medians, and triangles represent individual biological replicates.

Consistent with our prior observation that MAHS-expressing ASCs exhibit increased alkaline phosphatase staining during osteogenic induction [8], 11 targets from the Ossification list in the Harmonizome GeneRIF Biological Term Annotations dataset were significantly upregulated (Figure 2) [27,28]. Among the most strongly upregulated genes were *SERPINF1* (log_2_FC = 6.1) and *COL1A1*/*COL1A2* (log_2_FC = 2.4 and 2.3, respectively), which encode critical bone matrix structural components [40,41]. *ALPL* (log_2_FC = 2.9), another established marker of early osteoblast differentiation, was robustly induced [42]. Additional upregulated genes included *BMP1* (log_2_FC = 1.0) and *PDGFRB* (log_2_FC = 2.5), both of which influence osteogenic differentiation through extracellular matrix (ECM) remodeling [43,44]. As an additional functional measure of osteogenic potential, matrix calcium content was assessed at DIV14 and DIV20 to capture early/intermediate and later stages of in vitro differentiation (Figure 3). At DIV14, calcium accumulation remained modest, consistent with an early/intermediate differentiation state [16]; however, MAHS-expressing ASCs exhibited significantly greater matrix calcium content than AcGFP1 ASCs under osteogenic conditions. By DIV20, osteogenic induction produced robust calcium accumulation in both ASC lines, with MAHS-expressing ASCs maintaining higher matrix calcium content than AcGFP1 ASCs. These matrix mineralization data are consistent with our prior alkaline phosphatase findings and with the observed upregulation of osteogenesis-associated transcripts.

Given the known roles of Wnt/β-catenin and YAP signaling in adipogenic and osteogenic fate decisions, we next examined the expression of associated genes [45,46,47,48,49]. We thus combined Wnt/β-catenin target genes from the Stanford Wnt/Beta-catenin Signaling Targets list and YAP target genes from the YAP Harmonizome GeneRIF Biological Term Annotations dataset [27,28,50]; comparing our DEG list against this target set identified 7 significantly upregulated and 3 significantly downregulated genes (Figure 2). Several pro-osteogenic or canonical Wnt targets were upregulated, including *WISP2* (log_2_FC = 6.0), *MITF* (log_2_FC = 3.5), *POSTN* (log_2_FC = 2.3), and *MMP2* (log_2_FC = 2.1), suggesting a reinforcement of osteogenic commitment [51,52,53,54]. Additionally, the negative Wnt modulator *DKK3* (log_2_FC = −0.9) was downregulated (but below the predefined DEG fold-change threshold), potentially counteracting Wnt inhibition, while the Wnt activator *MET* (log_2_FC = −1.6) was also downregulated [55,56]. Within the YAP signaling pathway, the Wnt/YAP regulatory kinase *CSNK1D* (log_2_FC = 0.9) was modestly upregulated (but below the predefined DEG fold-change threshold), while the canonical YAP target *AREG* (log_2_FC = −2.4) was significantly downregulated, consistent with reduced YAP-dependent transcriptional output [57,58]. Further, supporting fluorescence imaging revealed a significant reduction in nuclear-to-cytoplasmic β-catenin (Figure S1) and YAP (Figure S2) ratios in MAHS-expressing ASCs.

Finally, given the importance of cellular metabolism in ASC fate, we analyzed gene expression changes against the Glycolytic Process and Mitochondrion lists in the Harmonizome GeneRIF Biological Term Annotations dataset, as well as the Metabolism list in the Harmonizome GA Gene Disease Associations dataset [27,28]. Twenty-four genes were significantly upregulated and 7 genes were significantly downregulated out of 646 genes in the aggregate list, consistent with a broad shift in energy handling in MAHS-expressing ASCs (Figure 2). Several crucial regulators of glycolysis were suppressed, including those responsible for glycolytic and metabolic flux such as *HK2* (log_2_FC = −2.0) and *PDK1* (log_2_FC = −1.2) [59,60,61]. *SLC2A1* (log_2_FC = −2.1), which encodes the glucose transporter GLUT1, was also downregulated, suggesting reduced glucose uptake capacity [62]. Among the most strongly downregulated genes was *GCKR* (log_2_FC = −5.2), a negative regulator of glucokinase in the liver; its suppression may indicate disrupted glucose sensing and lipid metabolism [63]. Conversely, *SLIT3* (log_2_FC = 4.6) and *ACE* (log_2_FC = 2.5) were among the most significantly upregulated metabolic genes, potentially implicating signaling pathways related to osteogenesis and glucose metabolism [64,65]. Mitochondria-associated genes also showed widespread upregulation, including strong induction of *PLA2G2A* (log_2_FC = 10.2), *CLU* (log_2_FC = 3.2), *RSAD2* (log_2_FC = 4.8), and *SPATA18* (log_2_FC = 3.1), which collectively suggest enhanced mitochondrial remodeling and stress adaptation [66,67,68,69]. Several key metabolic regulators, including *OAS1* (log_2_FC = 2.6) and *RARRES3* (log_2_FC = 2.8), were also upregulated, reflecting changes to lipid synthesis and mitochondrial regulation [70,71]. *CDKN1A* (log_2_FC = 1.7), a p53-inducible cell cycle inhibitor implicated in mitochondrial regulation, was also elevated, potentially supporting a connection between metabolic stress and altered cell cycle dynamics in MAHS-expressing ASCs [72]. Notably, *SLC25A27* (log_2_FC = 4.7), while not on the Harmonizome gene list, was significantly upregulated and encodes the mitochondrial uncoupling protein UCP4 that transports protons across the inner mitochondrial membrane and is a negative regulator of mitochondrial membrane potential (ΔΨ_m_) [73,74]. Further, several mitochondrial genes involved in metabolic homeostasis or maintenance of membrane potential were downregulated, including *NRP1* (log_2_FC = −1.7), *EHHADH* (log_2_FC = −1.3), and *NPC1* (log_2_FC = −1.1) [75,76,77]. The G-protein-coupled receptor *ADORA1* was also strongly upregulated (log_2_FC = 3.4) and may contribute to changes in adenosine and cAMP-related signaling pathways [78]. Furthermore, several genes associated with upregulation of the ubiquitous secondary messenger cyclic guanosine monophosphate (cGMP) were strongly upregulated, including *PRKG2*, *PRKAA2*, *NPR1*, *GUCY1A2*, and *NPR3*, while other related genes including *PRKCD* and *PRKCI* were weakly downregulated (Figure 2) [79,80,81,82]. Together, these findings suggest a broad reprogramming in the metabolic landscape of MAHS-expressing ASCs that may contribute to the observed impairment of adipogenesis and enhancement of osteogenesis.

### 2.2. MAHS Expression Reduces ATP Levels

**Figure 4:**
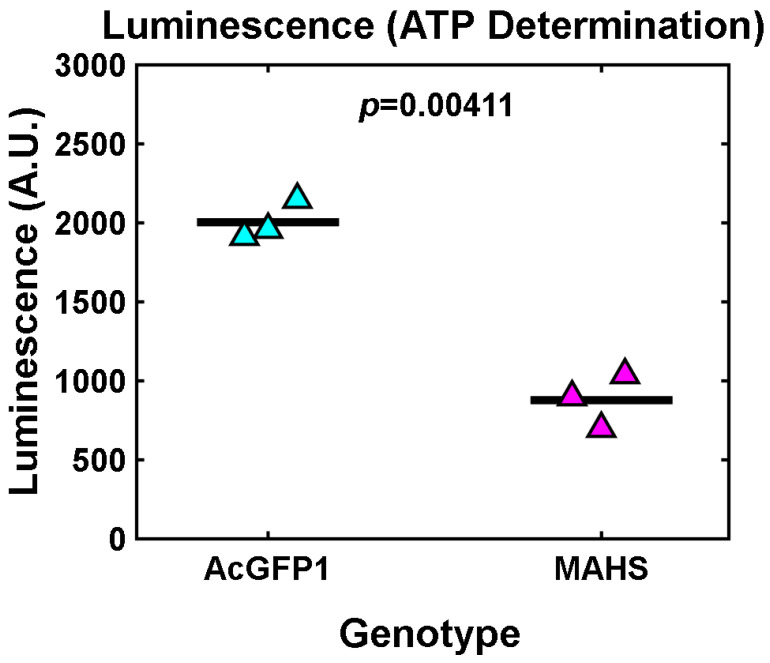
ATP determination. Quantification of luminescence resulting from the firefly luciferase reaction as a measure of ATP levels in AcGFP1- and MAHS-expressing ASCs (two-sample *t*-test, *n* = 3). Black lines represent means, and triangles represent individual biological replicates. The denoted *p*-value indicates the pairwise difference in luminescence readings between the genotypes.

Next, we indirectly quantified cellular energy levels in ASCs by measuring ATP concentration using a luciferin-based assay (Figure 4) [83]. Luminescence intensity was significantly lower in MAHS-transgenic ASCs than in AcGFP1-transgenic ASCs. These data show that MAHS expression decreases ASC cellular energy levels, potentially from ΔΨ_m_ disruption or the observed downregulation of genes associated with pyruvate enzymes and glucose metabolism and transport (e.g., *SLC2A1*, *HK2*, and *PDK1*) [84,85,86].

We further assessed the effect of MAHS expression on ΔΨ_m_ in ASCs. Tetramethylrhodamine methyl ester (TMRM) is a cell-permeable fluorescent dye that accumulates in mitochondria with intact membranes in a membrane potential-dependent manner [87]. Here, we used TMRM fluorescence intensity as a semiquantitative indicator of ΔΨ_m_, whereby higher fluorescence intensity corresponds to higher mitochondrial polarization (Figure S3) [88]. The fluorescence intensity of TMRM was significantly lower in MAHS-transgenic ASCs than in AcGFP1-transgenic ASCs. We did not detect any change in percent area coverage of TMRM in MAHS-transgenic ASCs (Figure S4), consistent with our prior results on mitochondrial morphology [8]. These data are consistent with MAHS expression reducing ΔΨ_m_ without altering overall mitochondrial volume or morphology.

### 2.3. MAHS Expression Reduces cAMP Levels

**Figure 5:**
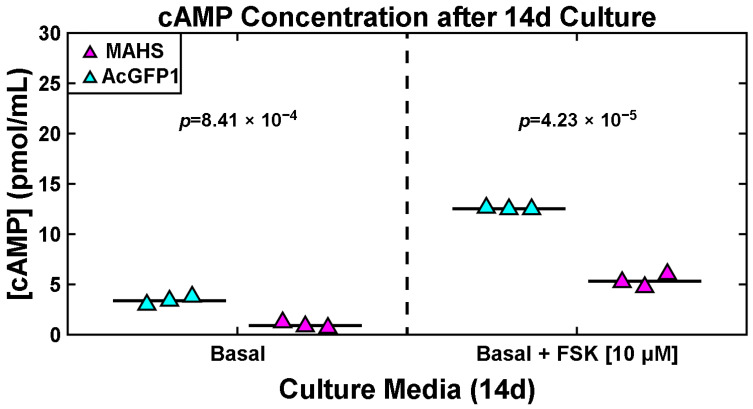
cAMP concentration. Quantification of mean cAMP concentration in AcGFP1- and MAHS-expressing ASCs with and without forskolin (FSK) supplementation (2-way ANOVA, Tukey’s HSD, *n* = 3). Black lines represent means and triangles represent individual biological replicates. The denoted *p*-value indicates the pairwise difference in cAMP concentration between the genotypes under each specific culture media condition.

Given the importance of cAMP signaling in ASC differentiation and the modified expression of several cAMP-related genes, we next aimed to assess the effect of MAHS expression on cAMP levels [16,17]. We quantified total cAMP concentration using a competitive ELISA assay, following 14 days of culture in basal media or media supplemented with the adenylyl cyclase (AC) activator forskolin (FSK) (Figure 5) [89]. Without FSK supplementation, total cAMP levels were significantly lower in MAHS-transgenic ASCs than in AcGFP1-transgenic ASCs, suggesting a baseline cAMP deficit. With FSK treatment, total cAMP levels increased in both AcGFP1 and MAHS-expressing ASCs, although cAMP levels in MAHS-expressing ASCs remained lower than in AcGFP1-transduced cells. These data show that MAHS expression diminishes cAMP levels. Given the importance of *ADORA1* and *PDE2A* as cAMP regulators, we further performed qPCR on these specific gene targets and found partially concordant results with the bulk sequencing. *ADORA1*, which was significantly upregulated (log_2_FC = 3.4; *p*_adj_ = 0.014) in the sequencing data, showed a concordant increase with qPCR that did not reach statistical significance (log_2_FC = 1.6; *p* = 0.106). While *PDE2A*, a promoter of cAMP hydrolysis, was not significantly downregulated in RNA sequencing analysis (log_2_FC = −2.1; *p*_adj_ = 0.28), qPCR showed a larger decrease that reached statistical significance (log_2_FC = −4.6; *p* = 0.0124) in MAHS-expressing ASCs (Figure S5) [90]. These results show directionally consistent changes in *ADORA1* and *PDE2A* expression across assays, but should be interpreted with appropriate caution given the differences in methods and statistical approach between RNAseq and qPCR [91].

As cAMP and cGMP pathways may be antagonistically regulated through shared phosphodiesterase (PDE) activity [92], we also measured cGMP levels using a competitive ELISA assay. ASCs were assessed at baseline and following 1 h treatment with the soluble guanylate cyclase (sGC) stimulator and indirect cGMP activator Riociguat at concentrations of 0.1 µM (Figure S6). No significant differences in cGMP levels were observed at baseline or following 0.1 µM Riociguat treatment.

### 2.4. Forskolin and Compound C Increase Lipid Deposition in MAHS-Expressing ASCs

**Figure 6:**
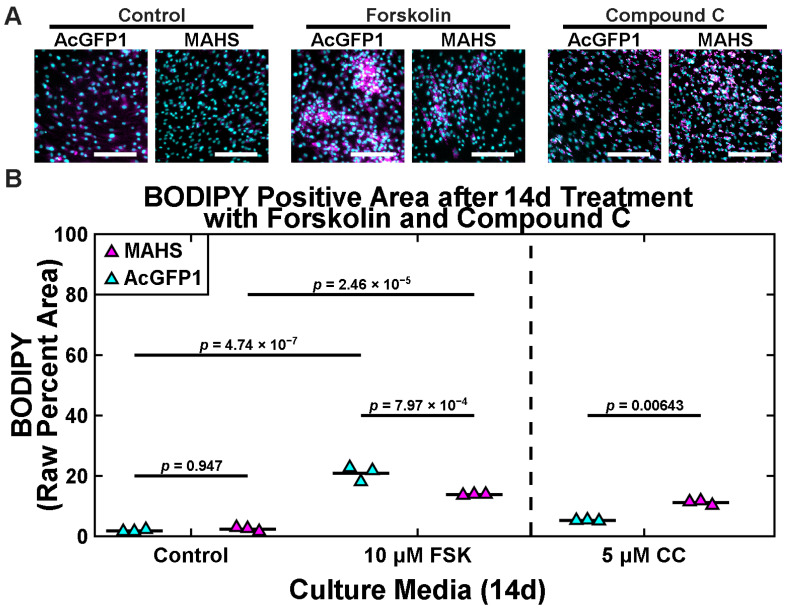
Adipogenic differentiation in the absence of adipogenic media. (**A**) Representative images of AcGFP1- (control) and MAHS-expressing ASCs 14 d treatment with 10 µM forskolin or 5 µM Compound C. Images are shown with a colorblind-accessible false coloration. Scale bars are 250 µm. (**B**) Quantification of mean BODIPY-positive area in AcGFP1- and MAHS-expressing ASCs with and without supplementation with forskolin (FSK) (2-way ANOVA, Tukey’s HSD, *n* = 3) or Compound C (two-sample *t*-test, *n* = 3). Black lines represent means, and triangles represent individual biological replicates. The denoted *p*-values indicate the pairwise differences in BODIPY-positive area between the genotypes under each specific culture media condition.

The above data suggest that changes to ASC metabolism, especially glycolysis, and cAMP signaling may play a role in the observed reduction in adipogenesis. To assess whether pharmacologic modulation of cAMP signaling or cellular metabolic state could promote adipogenic lipid accumulation in MAHS-expressing ASCs, we quantified the positive area of the neutral lipid droplet marker BODIPY following 14 days of culture in routine media supplemented with the adenylyl cyclase (AC) activator forskolin (FSK) or the non-selective AMPK inhibitor Compound C (Figure 6) [93,94,95]. BODIPY-positive area was significantly lower in MAHS-expressing ASCs relative to control ASCs following 10 µM FSK, though an increase in MAHS-expressing ASCs supplemented with FSK relative to routine culture media only was observed. Further, Compound C supplementation produced increased BODIPY-positive area in MAHS-expressing ASCs over control ASCs.

## 3. Discussion

Overall, this study shows that MAHS expression in ASC52telo cells is associated with reduced adipogenic differentiation, enhanced osteogenic differentiation, and coordinated changes in metabolic and signaling pathways consistent with altered lineage bias.

To broadly characterize the impact of MAHS expression on ASC biology, we first performed transcriptomic profiling under routine culture conditions (Figure 1 and Figure 2). The impaired adipogenic but enhanced osteogenic differentiation in MAHS-expressing ASCs observed in prior work and here was reflected in a gene expression profile suggestive of partial but insufficient adipogenic activation [8]. Several key regulators, including *HSD11B1*, *ADORA1*, and *SREBF1*, were upregulated, suggesting that a core adipogenic transcriptional framework remains partially intact [29,30,31]. However, strong suppression of *SSTR1* and *ENHO*, genes involved in metabolic and hormonal support of adipogenesis, suggests additional impairment to the cellular response to differentiation signals [36,37]. In contrast, osteogenic programming appeared to be actively reinforced. Consistent with our observations of enhanced osteogenic differentiation and increased mineralized calcium deposition, Harmonizome-annotated ossification genes were significantly upregulated [8,27,28]. *SERPINF1* is particularly notable because it encodes pigment epithelium-derived factor (PEDF), a secreted factor implicated not only in osteogenesis but also in the regulation of angiogenesis, fibrosis, and inflammation [96,97], making it a plausible candidate link between MAHS-associated osteogenic bias and broader signaling alterations. The upregulation of *BMP1* and *PDGFRB*, both involved in ECM remodeling and osteoinductive signaling, further supports a shift toward an osteogenic transcriptional state [43,44]. Together, these findings suggest that MAHS expression not only impairs adipogenic commitment but simultaneously promotes osteogenic lineage bias, potentially through coordinated alterations to ECM signaling and metabolic state.

The Wnt/β-catenin and YAP data further suggest that MAHS expression alters lineage-associated signaling, although these changes do not map cleanly onto simple pathway activation or inhibition. Transcriptomic profiling revealed upregulation of several canonical Wnt targets alongside downregulation of the Wnt inhibitor *DKK3*, collectively suggesting enhanced Wnt-associated signaling [51,52,53,54,55]. In parallel, transcriptomic changes within the YAP axis were notable, with modest upregulation of *CSNK1D* and suppression of *AREG*, pointing to partial attenuation of YAP transcriptional output [57,58]. Despite the upregulation of pro-osteogenic Wnt targets, fluorescence imaging showed reduced nuclear localization of both β-catenin (Figure S1) and YAP (Figure S2), indicating that downstream nuclear transduction of these pathways is dampened in MAHS-expressing ASCs. This is in contrast to the canonical understanding of β-catenin and YAP, where osteogenic differentiation (as seen in MAHS-expressing ASCs) is typically associated with increased nuclear localization. Accordingly, these data should be interpreted as evidence of altered, rather than simply activated or deactivated, signaling.

Metabolism and mitochondrial function play a critical role in directing mesenchymal stem cell fate, with adipogenesis favoring sustained glycolytic metabolism and osteogenesis typically involving a shift from glycolysis to oxidative phosphorylation as differentiation progresses [98,99]. In this context, the broad metabolic reprogramming observed in MAHS-expressing ASCs likely contributes to the shift in lineage bias. Downregulation of key glycolytic genes may indicate impaired glucose uptake and reduced glycolytic flux, both of which support lipid biosynthesis and the energy demands of adipogenic differentiation [59,61,62]. This may disproportionately affect adipogenic maturation, which relies heavily on glycolysis and efficient energy storage, while supporting a shift toward oxidative phosphorylation associated with osteogenesis [98,99].

Consistent with these findings, functional assays suggest that MAHS-expressing ASCs may have a lower baseline mitochondrial membrane potential (ΔΨ_m_) than control ASCs (Figure S3); if true, this may be due to membrane disruption caused by MAHS insertion or downstream expression changes. Consistent with this, we observed a lower baseline ATP measurement in MAHS-expressing ASCs (Figure 4). Disruption of ATP production may also impact secondary messenger systems involved in metabolism and stem cell fate decisions [100]. In particular, cAMP signaling, which depends on ATP availability for adenylyl cyclase activity, was markedly impaired in MAHS-expressing ASCs (Figure 5) [101]. Although forskolin supplementation partially elevated cAMP levels, it failed to fully rescue the deficit, consistent with the hypothesis of decreased ATP availability being a bottleneck for cAMP synthesis in MAHS-expressing cells. To test whether increasing cAMP is a potential mechanism for promoting adipogenesis in MAHS-expressing cells, we assessed adipogenic capacity by quantifying BODIPY-positive lipid accumulation under routine culture and after forskolin treatment. MAHS-expressing ASCs exhibited significantly reduced lipid content at baseline, consistent with only a modest increase with forskolin supplementation, reinforcing the role of disrupted cAMP signaling in this phenotype (Figure 6). Of additional interest, in prior work we used adipogenesis media containing IBMX, a non-selective PDE inhibitor [8], which results in reduced degradation of cAMP and increased cAMP levels [90]. However, our current transcript data shows reduced expression of *PDE2A* (Figure S5), potentially as a compensatory mechanism for lower cAMP levels, which may limit the effectiveness of IBMX. Together, these findings are consistent with impaired cAMP production in MAHS-expressing cells, potentially secondary to reduced ATP availability. Because cAMP and cGMP pathways are often coregulated via shared phosphodiesterase activity and several cGMP-associated transcripts were altered, direct cGMP measurements did not demonstrate a clear baseline difference, so the role of cGMP signaling remains unresolved.

To determine whether metabolic signaling modulation could alter this phenotype, we next treated cells with Compound C (Figure 6). Strikingly, lipid accumulation increased significantly in MAHS-expressing ASCs, even surpassing levels in control cells. One possible mechanism is Compound C-mediated inhibition of AMPK, as activation of AMPK in MAHS-expressing cells would be consistent with the observed reduction in ATP levels [102]. However, while Compound C is commonly used as an AMPK inhibitor [103], it is known to have inhibitory effects on a number of other kinases and signaling pathways, complicating analysis [95,104,105]. Of specific relevance to osteogenesis and adipogenesis, BMP signaling [106,107,108] is inhibited by Compound C, as well as other kinases [104,105]. Compound C restored adipogenic capacity, reinforcing the broader trend of metabolic and signaling reprogramming in response to exogenous MAHS expression. Together, these findings indicate that the adipogenic defect in MAHS-expressing ASCs can be partially rescued, although the mechanism remains unresolved.

While these findings offer an initial view of how MAHS expression reprograms ASC metabolism, signaling, and differentiation potential, several limitations remain. A core limitation of our study is related to the use of bulk RNAseq, which captures population-level transcriptional changes but cannot resolve cell-to-cell heterogeneity or determine whether transcript-level changes correspond to altered protein abundance or secreted factor production. Relatedly, although transcriptomic data point towards several candidate regulatory pathways, we did not comprehensively validate key transcription factors or protein expression by targeted qPCR, Western blotting, or other techniques. Accordingly, protein-level validation of selected adipogenic, osteogenic, metabolic, and signaling regulators remains an important limitation of the present study. Another limitation is the use of ASC52telo cells, an hTERT-immortalized ASC line. Although ASC52telo was initially reported to retain key mesenchymal marker expression and multilineage differentiation capacity [109], subsequent studies have shown that this line can differ from primary ASCs, particularly with respect to adipogenic differentiation efficiency and insulin- and cAMP-dependent signaling [110,111,112]. These reported differences are relevant to the present study due to MAHS-associated phenotypes concerning adipogenic differentiation and cAMP signaling. Thus, while ASC52telo provides a reproducible model for initial mechanistic studies and has supported robust adipogenic differentiation in our prior work [8,113], validation in primary ASCs remains necessary. Similarly, chondrogenic differentiation was not quantitatively assessed in the present study, which is a limitation given that osteogenic and chondrogenic differentiation arise from related skeletal-lineage programs and are regulated by overlapping signaling pathways, including BMP/TGFβ, Wnt/β-catenin, and SOX9/RUNX2 [114,115]. Thus, the extent to which MAHS affects broader skeletal lineage specification, including chondrogenic commitment, remains unresolved. Moreover, in vivo studies are needed to evaluate how MAHS expression and subsequent changes to ASC stemness and metabolism translate in the context of tissue regeneration or repair, and cytotoxicity assessments will be essential to determine the safety of MAHS expression in physiologically relevant cellular environments. Finally, while this work identifies several promising avenues for altering the metabolic and differentiation phenotype of MAHS-expressing ASCs, future studies should assess whether long-term modulation of these pathways can restore full multipotency or whether the differentiation bias imposed by MAHS is partially irreversible. Despite these limitations, our results provide a foundation for understanding how mitochondrial disruption and energetic stress reshape ASC function and highlight key regulatory nodes that may serve as targets for restoring lineage plasticity in metabolically impaired stem cells.

## 4. Materials and Methods

### 4.1. Routine Cell Culture

All cell types were routinely cultured at 37 °C and 5% CO_2_. hTERT-immortalized adipose-derived mesenchymal stem cell (ASC52telo; ATCC SCRC-4000, Manassas, VA, USA) parental and transgenic lines were cultured in Dulbecco’s Modified Eagle Medium/Nutrient Mixture F-12 (Thermo Fisher Scientific #11320082, Waltham, MA, USA) supplemented with fetal bovine serum (10%), penicillin-streptomycin (1%), and amphotericin B [25 ng/mL]. Cells were regularly passaged at ~ 80% confluence, passage-3 cells were used for transduction, and all experiments were performed between 18 and 25 passages from transduction.

### 4.2. Cloning and Lentiviral Transduction

For the current studies, we used ASC52telo-expressing AcGFP1 (as control) or MAHS-AcGFP1 (referred to as “MAHS” throughout the manuscript for brevity) we previously established as described in our prior work [8], including validation of MAHS localization to the mitochondria. Briefly, AcGFP1 (Addgene #54705; Addgene, Watertown, MA, USA) and MAHS fusion (Addgene #90034) were cloned into a lentiviral transfer vector (pCDH-CMV-MCS-EF1α-Puro; Systems Biosciences, Palo Alto, CA, USA). Lentivirus was produced in HEK293TN cells via triple transfection with a packaging plasmid (psPAX2; Addgene #12260), an envelope plasmid (pMD2.G; Addgene #12259), and the respective transfer vector using Endofectin Max (GeneCopoeia, Rockville, MD, USA; #EF013). Viral supernatant was collected, filtered, and used to transduce ASC52telo cells, followed by puromycin selection to establish stable cell lines. After selection, cells were routinely checked to ensure maintenance of stable transgenic population.

### 4.3. RNA Sequencing

AcGFP1- and MAHS-expressing ASCs were seeded at a density of 50,000 cells per well and cultured for 24 h in routine culture media. Total RNA was isolated at each time point using the GeneJET RNA Purification Kit (K0731; Thermo Fisher Scientific, Waltham, MA, USA) according to manufacturer instructions. RNA concentrations of each sample were quantified with the SpectraDrop Micro-Volume Microplate reader (Molecular Devices, San Jose, CA, USA). Three separate cell platings were used as biological replicates.

Bulk RNA library preparation and sequencing were performed by Novogene (Novogene Corporation, Sacramento, CA, USA). Libraries were prepared using poly(A) enrichment and 150 bp paired-end reads were generated from Illumina platform sequencing. Quality-control filtering of raw sequencing data was used to remove adapter-containing reads, reads with >10% undetermined bases, and low-quality reads with >50% of bases with Q scores ≤ 5. HISAT2 was used to align filtered reads to the human reference genome (GRCh38). Gene expression levels were normalized and reported as raw read counts and fragments per kilobase of transcript per million mapped reads (FPKM).

Differentially expressed gene (DEG) analysis of log_2_(fold changes) was performed in R (Version 4.1.2) using DESeq2. The Benjamini–Hochberg false discovery rate was used to identify significantly differentially expressed genes (*p*_adj_ < 0.05), and only genes with |log_2_FC| ≥ 1 were considered for downstream analysis. Functional enrichment analysis of differentially expressed genes was conducted using Gene Ontology (GO). The DEG list is provided as Supplementary Data S1. Complete RNA-seq datasets generated and analyzed during the current study are available in the NCBI Gene Expression Omnibus (GEO) repository under accession number GSE325200.

Additionally, we assessed differential gene expression in reference to targeted gene lists of specific relevance. Due to the importance of metabolism, we merged the Glycolytic Process and Mitochondrion lists in the Harmonizome GeneRIF Biological Term Annotations dataset, as well as the Metabolism list in the Harmonizome GA Gene Disease Associations dataset, and compared them to our DEG analysis [27,28]. Similarly, we compared our DEG list to Wnt and YAP target gene lists from the Stanford Wnt/Beta-catenin Signaling Targets list and the YAP Harmonizome GeneRIF Biological Term Annotations dataset, given the importance of Wnt and YAP signaling in ASC fate determination [27,28,50].

### 4.4. ATP Determination Measurement

AcGFP1- and MAHS-expressing ASCs were seeded at a density of 50,000 cells per well in a 24-well plate and allowed to adhere for 24 h in routine culture media. After 24 h, cellular ATP levels were assessed using the ATP Determination Kit following the manufacturer’s protocol (A22066; Thermo Fisher Scientific, Waltham, MA, USA). ATP-dependent bioluminescence was measured using a SpectraMax M2 Microplate Reader (Molecular Devices, San Jose, CA, USA). Data are reported as relative luminescence units (RLU) compared to controls. Three separate cell platings were used as biological replicates.

### 4.5. Mitochondrial Membrane Potential Measurement

AcGFP1- and MAHS-expressing ASCs were seeded at a density of 10,000 cells per well in a 24-well plate and allowed to adhere for 24 h in routine culture media. After 24 h, cells were subsequently labeled using Tetramethylrhodamine, Methyl Ester (TMRM; HY-D0984; MedChemExpress, Monmouth Junction, NJ, USA) to assess mitochondrial membrane potential. A 5 mM TMRM stock solution was prepared by dissolving 1 mg TMRM in 525 µL dimethyl sulfoxide (DMSO; MFCD00002089; Gaylord Chemical Company, Covington, LA, USA). The stock solution was diluted in PBS to a final working concentration of 10 µM. Cells were aspirated of media and 1 mL of the TMRM working solution was added per well. Cells were incubated at room temperature in the dark for 40 min, followed by three washes with routine culture medium. Imaging was performed within two hours of staining using a DFC9000 GT sCMOS camera on a DMi8 inverted microscope (Leica Microsystems, Chicago, IL, USA). TMRM was imaged using a Texas Red filter set (Leica #15525310). A 25-position tile scan of a ~0.37 cm^2^ area was obtained for each sample with 1 µm steps to focus each image. Five separate cell platings were used as biological replicates. A custom MATLAB (version 2025a) script was used to quantify the fluorescence intensity of the TMRM label. For all analyses, cell-by-cell fluorescence intensity measurements were calculated for each sample. Representative images are shown with colorblind-accessible false colors of magenta/cyan/yellow according to published recommendations [116].

### 4.6. cAMP ELISA Assay

AcGFP1- and MAHS-expressing ASCs were seeded at a density of 50,000 cells per plate in 35 mm culture plates, with three replicates per plate and three biological replicates per condition. Cells were cultured for 14 days in routine culture media or routine culture media supplemented with 10 µM of the cAMP activator forskolin (FSK; HY-15371; MedChemExpress, Monmouth Junction, NJ, USA), with media changes every 3 days [16,117]. Intracellular cyclic AMP (cAMP) levels were measured using a Cyclic AMP ELISA Kit (581001; Cayman Chemical, Ann Arbor, MI, USA) following the manufacturer’s protocol for acetylated samples. Absorbance values corresponding to enzymatic reaction product intensity were measured at 405 nm using a SpectraMax M2 Microplate Reader (Molecular Devices, San Jose, CA, USA). The resulting %B/B_0_ data, inversely proportional to cAMP concentrations in the sample, were fit to a four-parameter logistic (4PL) regression curve to determine cAMP concentrations in pmol/mL.

### 4.7. cGMP ELISA Assay

AcGFP1- and MAHS-expressing ASCs were seeded at a density of 50,000 cells per well in 24-well culture plates, with three replicates per plate and three biological replicates per condition. At 24 h after plating, cells were cultured for 1 h in routine culture media or routine culture media supplemented with 0.1 µM Riociguat (BAY 632521; HY-14779; MedChemExpress, Monmouth Junction, NJ, USA). Intracellular cyclic guanosine monophosphate (cGMP) levels were measured using a Cyclic GMP ELISA Kit (581021; Cayman Chemical, Ann Arbor, MI, USA) following the manufacturer’s protocol for acetylated samples. Absorbance values corresponding to enzymatic reaction product intensity were measured at 410 nm using a SpectraMax M2 Microplate Reader (Molecular Devices, San Jose, CA, USA). The resulting %B/B_0_ data, inversely proportional to cGMP concentrations in the sample, were fit to a four-parameter logistic (4PL) regression curve to determine cGMP concentrations in pmol/mL.

### 4.8. Adipogenic Differentiation

AcGFP1- and MAHS-expressing ASCs were seeded at a density of 10,000 cells per well in a 48-well plate and cultured for 14 days in routine culture media supplemented with 250 µM 3-isobutyl-1-methylxanthine (IBMX; 284550; Tocris Bioscience, Bristol, UK) or 10 µM forskolin (FSK; HY-15371; MedChemExpress, Monmouth Junction, NJ, USA), with media changes every 3 days. After 14 days, samples were fixed, labeled with BODIPY 493/503 (D3922; Molecular Probes, Eugene, OR, USA), and imaged as described in prior work [8]. Three separate cell platings were used as biological replicates. Adipogenic expression was quantified as the percent of cellular BODIPY-positive area using a previously described custom MATLAB script [8], with quantification based on cell counts from 25 positions per sample. BODIPY was imaged using a GFP filter set (Leica #15525314). Representative images are shown with colorblind-accessible false colors of magenta/cyan/yellow according to published recommendations [116].

### 4.9. Osteogenic Differentiation and Matrix Assessment

Osteogenic differentiation was initiated as previously described [8]. In brief, AcGFP1- and MAHS-expressing ASCs were prepared for differentiation by seeding 10,000 cells/well in a 48-well plate with routine culture media. Medium was substituted with osteogenic differentiation media (osteocyte differentiation tool; ATCC PCS-500-052, Manassas, VA, USA) after 48 h. Media were changed every 4 days. At 14 and 20 days of differentiation, cells were lysed in radio-immunoprecipitation (RIPA) buffer (1% NP40, 0.5% sodium deoxycholate, 0.1% sodium dodecyl sulfate, phosphate-buffered saline (PBS), all Sigma-Aldrich) and assayed for calcium content as per Davis et al. [118]. Calcium crystals that remained on the plate after lysis were dissolved with 1 N HCl and collected. Calcium and HCl lysates were measured with Arsenazo III (Genzyme, Cambridge, MA, USA) at 655 nm using an iMark microplate reader (BioRad, Hercules, CA, USA), and compared to a CaCl_2_ standard. Calcium content was normalized to total protein, determined via Lowry assay (BioRad) at 750 nm after 15 min of shaking, using a bovine serum albumin standard curve.

### 4.10. Wnt and Hippo Immunocytochemistry

AcGFP1- and MAHS-expressing ASCs were seeded at a density of 10,000 cells per well in a 24-well plate and cultured for 24 h in routine culture media. After 72 h, cells were fixed for 5 min with 4% paraformaldehyde (T353500; ThermoFisher Scientific, Waltham, MA, USA) in PBS and permeabilized for 5 additional minutes with 0.25% Triton X-100 (BP151500; ThermoFisher Scientific, Waltham, MA, USA) in PBS. Samples were incubated overnight at 4 °C in a primary antibody solution of [1:500] YAP (D8H1X) XP (R) (14074; Cell Signaling Technology, Danvers, MA, USA) or β-catenin (b-catenin (H-1)) (sc-133240; Santa Cruz Biotechnology, Dallas, TX, USA). After 24 h, samples were washed three times with PBS and incubated for an additional 24 h at 4 °C in a secondary solution of [1:750] Goat anti-Rabbit IgG Secondary Antibody, Alexa Fluor 555 (A-21428; Thermo Fisher Scientific, Waltham, MA, USA) and [1:750] DyLight Phalloidin 650 (12956; Cell Signaling Technology, Danvers, MA, USA) in 1× DAPI (D1306; Invitrogen, Waltham, MA, USA). After 24 h, samples were washed three times with PBS and imaged as described above. DAPI, Alexa Fluor 555, and Dylight 650 were imaged with DAPI Long Pass (Leica #15525301), Texas Red (Leica #15525310), and Y5 (Leica #15525312) filter sets, respectively. Three separate cell platings were used as biological replicates. A custom MATLAB script was written to quantify YAP, TAZ, and β-catenin fluorescence intensity in the cell nuclear and cytoplasmic regions, as well as the ratio of nuclear to cytoplasmic intensity in each cell. For all analyses, fluorescence intensity measurements were averaged across 25 positions per sample. Representative images are shown with colorblind-accessible false colors of magenta/cyan/yellow according to published recommendations [116].

### 4.11. RT-qPCR

AcGFP1- and MAHS-expressing ASCs were seeded at a density of 50,000 cells per well in a 24-well plate. For metabolic gene expression analysis, cells were cultured in basal media for 24 h. For adipogenic gene expression markers, cells were cultured in either basal or adipogenic differentiation media (DMEM/F-12 50-50 base media (15-090-CM; Corning, Corning, NY, USA), fetal bovine serum (3%, A5256701; Gibco, Billings, MT), biotin [200.56 mM] (B4501; Sigma–Aldrich, St. Louis, MO, USA), IBMX [100 mM] (284550; Tocris Bioscience, Bristol, UK), indomethacin [20 mg/mL] (SC-200503; Santa Cruz Biotechnology, Dallas, TX, USA), insulin [5 mg/mL] (100-11; PeproTech, Cranbury, NJ, USA), dexamethasone [1 mM] (50-02-2; Sigma–Aldrich, St. Louis, MO, USA), and d-calcium pantothenate [671.53 mM] (AC416750000; ThermoFisher Scientific, Riverside, CA, USA) for 14 or 21 days. Three separate cell platings per condition were used as biological replicates.

Total RNA was isolated at each timepoint using the GeneJET RNA Purification Kit (K0731; Thermo Fisher Scientific, Waltham, MA, USA) according to manufacturer instructions. RNA concentrations of each sample were quantified with the SpectraDrop Micro-Volume Microplate reader. Gene expression was assessed using one-step RT-qPCR with the qPCRBIO SyGreen 1-Step Detect Lo-ROX Kit with 20× RTase (PB25.11-01; PCR Biosystems, London, UK) with 270 ng of total RNA per reaction. Samples were amplified on the MIC qPCR Cycler (Bio Molecular Systems, Upper Coomera, Australia) and melt curve analysis was performed from 72 °C to 95 °C to verify amplification specificity.

Gene expression was normalized to the housekeeping gene TATA-box binding protein (*TBP*), and relative fold gene expression was calculated for each sample using the 2^−ΔΔCt^ method [119]. Primer targets included metabolic genes related to cAMP signaling (e.g., phosphodiesterase [*PDE2A*], adenosine receptor 1 [*ADORA1*]). All primer sequences are listed in Table 1.

### 4.12. Statistics

For mitochondrial membrane potential measurement and ATP determination, statistical comparisons between groups were performed using a two-sample *t*-test with a significance threshold of α = 0.05 (MATLAB). For cAMP and cGMP ELISA experiments, statistical comparisons between groups were performed using 2-way analysis of variance (2-way ANOVA) to assess the effects and interactions of cell genotype and culture media, followed by Tukey’s honestly significant difference (HSD) post hoc test for pairwise comparisons with a significance threshold of α = 0.05 (MATLAB). For adipogenic differentiation studies and Wnt signaling assays, statistical analyses were performed using 2-way analysis of variance and Tukey’s HSD and visually displayed as previously described [8]. RT-qPCR comparisons were performed on relative expression values from the ΔΔC_t_ method using two-sample *t*-tests, with fold changes used for visualization. For osteogenic differentiation studies, statistical analysis was performed in GraphPad Prism (10.2.3., GraphPad Software Inc., San Diego, CA, USA) using Brown–Forsythe and Welch ANOVA tests assuming non-equal standard deviations followed by Dunnett’s post hoc test to correct for multiple comparisons. The datasets used for graphical representations and statistical analyses are available in the Supplementary Materials as Supplementary Data S2.

## Figures and Tables

**Table 1:** Human qPCR primer sequences used in these studies.

Gene	Direction	Sequence (5′ → 3′)	Reference
*TBP*	FWD	TGTATCCACAGTGAATCTTGGTTG	OriGene HP206762 [120]
*TBP*	REV	GGTTCGTGGCTCTCTTATCCTC	OriGene HP206762 [120]
*PDE2A*	FWD	GCTGGTGAACAAGATCAATGGGC	OriGene HP206257 [121]
*PDE2A*	REV	GCTGCGATACTGAGCCTCATTC	OriGene HP206257 [121]
*ADORA1*	FWD	ATTGCTGTGGACCGCTACCTCC	OriGene HP200623 [122]
*ADORA1*	REV	CGCACTCAGATTGTTCCAGCCA	OriGene HP200623 [122]

## Data Availability

The RNA-seq datasets generated and analyzed during the current study are available in the NCBI Gene Expression Omnibus (GEO) repository under accession number GSE325200 (https://www.ncbi.nlm.nih.gov/geo/query/acc.cgi?acc=GSE325200 (accessed on 18 March 2026)). The other original contributions presented in this study are included in the article/Supplementary Material.

## Supplementary Materials

The following supporting information can be downloaded at: https://www.mdpi.com/article/10.3390/ijms27146289/s1.
