# Supplementary material for: Mitochondrial Abundant Heat Soluble (MAHS) Protein Expression Modulates Metabolic Dynamics in Human Adipose-Derived Stem Cells
Source: Int J Mol Sci. 2026 Jul 15;27(14):6289. doi: 10.3390/ijms27146289 (PMC13412036; doi:10.3390/ijms27146289)
Supplement: Supplementary file 1 [file ijms-27-06289-s001.zip › Supplemental Figures.pdf]

Supplemental Information

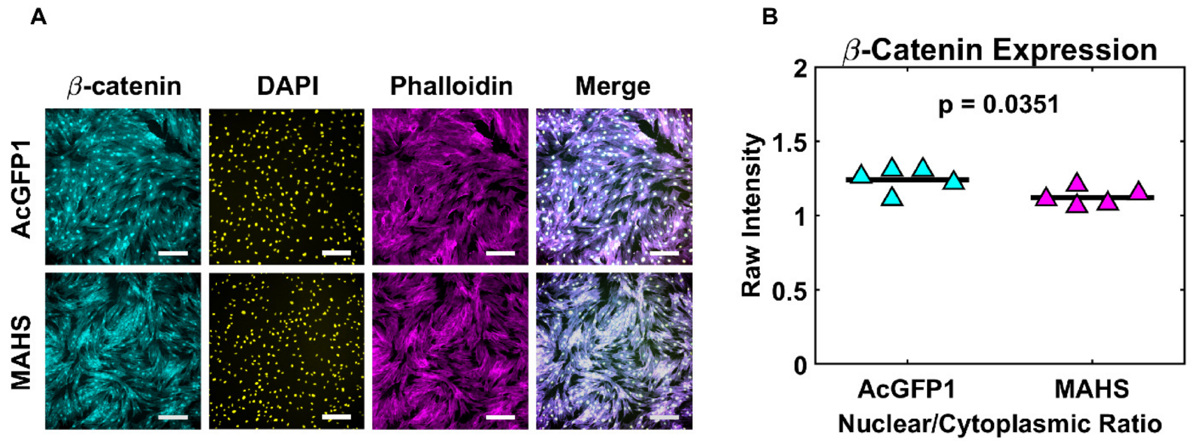

Figure S1. β-catenin expression. **(A)** Representative images of β-catenin expression in AcGFP1- (control) and MAHS-expressing ASCs under 24-hour basal culture media conditions. Images are shown with a colorblind accessible false coloration. Scale bars are 250 μm. **(B)** Nuclear/cytoplasmic ratio of β-catenin intensity (two-sample *t*-test, $n = 5$). Black lines represent means and triangles represent individual biological replicates. The denoted p-value indicates the pairwise difference in mean β-catenin intensity between the genotypes at each localization.

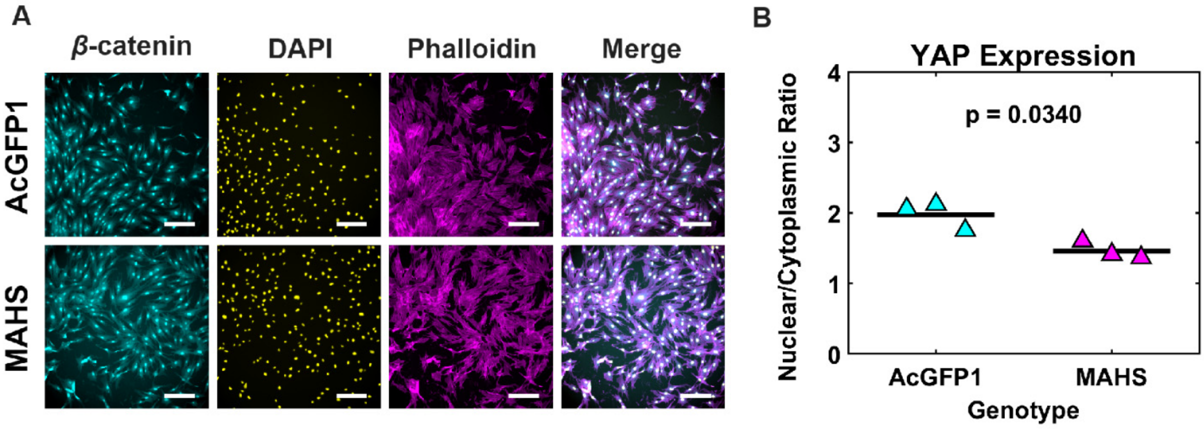

Figure S2. YAP expression. **(A)** Representative images of YAP expression in AcGFP1- (control) and MAHS-expressing ASCs under 24-hour basal culture media conditions. Images are shown with a colorblind accessible false coloration. Scale bars are 250 µm. **(B)** Nuclear/cytoplasmic ratio of YAP intensity (two-sample *t*-test, $n = 3$). Black lines represent means and triangles represent individual biological replicates. The denoted p-value indicates the pairwise difference in mean YAP intensity between the genotypes.

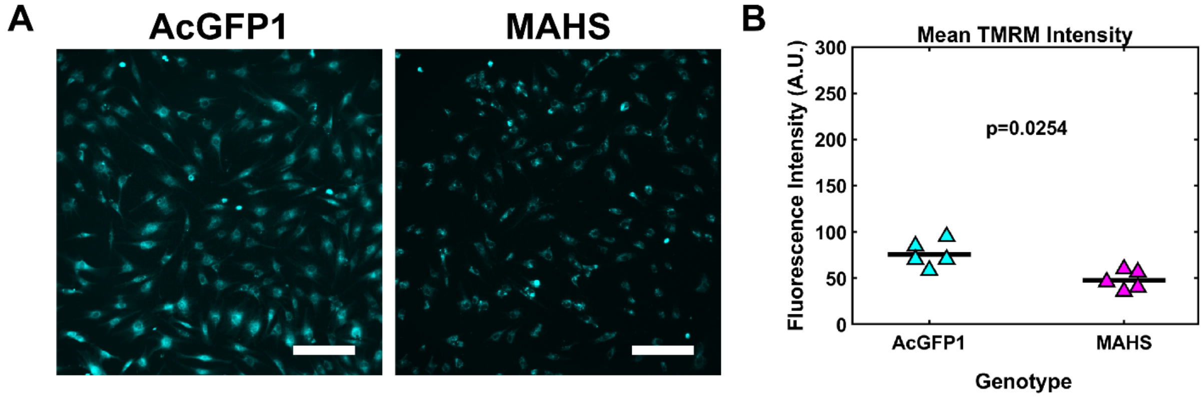

Figure S3. Mitochondrial membrane potential. **(A)** Representative images of ASC52telos transduced with either the AcGFP1 or MAHS transgene, labeled for mitochondrial membrane potential (TMRM, cyan). Images are shown with a colorblind accessible false coloration. Scale bars are 250 µm. **(B)** Quantification of mean TMRM fluorescence intensity in AcGFP1- and MAHS-expressing ASCs (two-sample *t*-test, n = 5). Black lines represent means and triangles represent individual biological replicates. The denoted p-value indicates the pairwise difference in mean TMRM intensity between the genotypes.

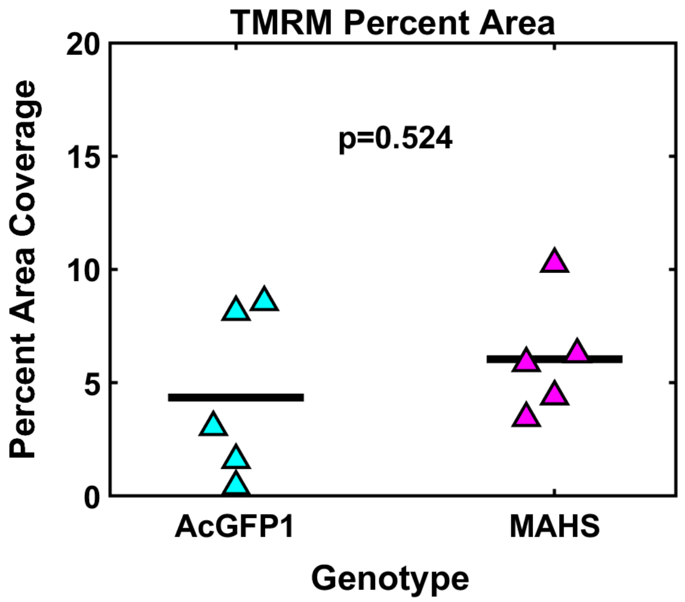

Figure S4. Percent area coverage of TMRM. Quantification of mean percent cell area coverage of TMRM in AcGFP1- and MAHS-expressing ASCs (two-sample *t*-test, $n = 5$). Black lines represent means and triangles represent individual biological replicates. The denoted p-value indicates the pairwise difference in mean TMRM percent area coverage between the genotypes.

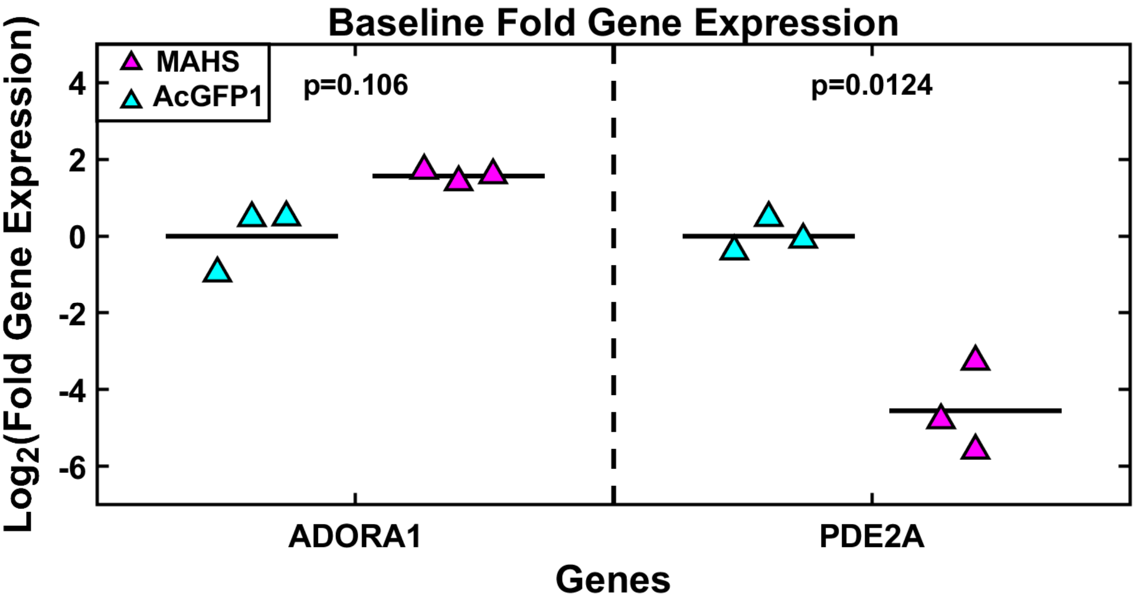

Figure S5. *ADORA1* and *PDE2A* gene expression. **(A)** Baseline fold gene expression of *ADORA1* and *PDE2A* in AcGFP1- (control) and MAHS-expressing ASCs under 24-hour basal culture media conditions. (two-sample *t*-test, $n = 3$). Black lines represent means and triangles represent individual biological replicates. The denoted p-value indicates the pairwise difference in mean baseline fold gene expression between the genotypes.

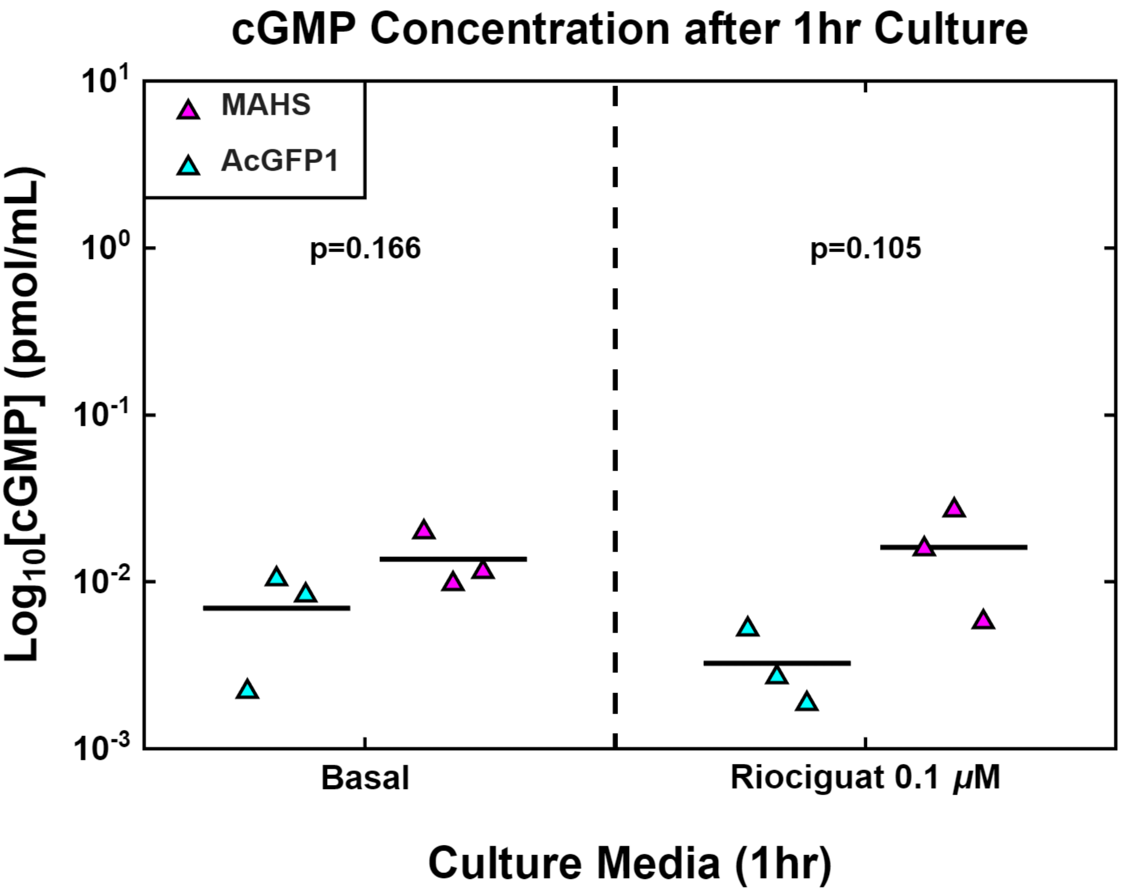

Figure S6. cGMP concentration. Quantification of mean cGMP concentration in AcGFP1- and MAHS-expressing ASCs with and without Riociguat supplementation (2-way ANOVA, Tukey's HSD, $n = 3$). The denoted p-value indicates the pairwise difference in $\log_{10}$(cGMP) concentration between the genotypes under each specific culture media condition.
